# Detection of Cell Types Contributing to Cancer From Circulating, Cell-Free Methylated DNA

**DOI:** 10.3389/fgene.2021.671057

**Published:** 2021-07-27

**Authors:** Megan E. Barefoot, Netanel Loyfer, Amber J. Kiliti, A. Patrick McDeed, Tommy Kaplan, Anton Wellstein

**Affiliations:** ^1^Department of Oncology, Lombardi Comprehensive Cancer Center, Georgetown University, Washington, DC, United States; ^2^School of Computer Science and Engineering, The Hebrew University of Jerusalem, Jerusalem, Israel; ^3^Department of Biochemistry and Molecular and Cellular Biology, Georgetown University, Washington, DC, United States; ^4^Department of Biostatistics, Bioinformatics, and Biomathematics, Georgetown University, Washington, DC, United States

**Keywords:** Cell-free DNA (cfDNA), cellular damage, circulating tumor DNA (ctDNA), deconvolution, liquid biopsy, tissue-of-origin, tumor microenvironment

## Abstract

Detection of cellular changes in tissue biopsies has been the basis for cancer diagnostics. However, tissue biopsies are invasive and limited by inaccuracies due to sampling locations, restricted sampling frequency, and poor representation of tissue heterogeneity. Liquid biopsies are emerging as a complementary approach to traditional tissue biopsies to detect dynamic changes in specific cell populations. Cell-free DNA (cfDNA) fragments released into the circulation from dying cells can be traced back to the tissues and cell types they originated from using DNA methylation, an epigenetic regulatory mechanism that is highly cell-type specific. Decoding changes in the cellular origins of cfDNA over time can reveal altered host tissue homeostasis due to local cancer invasion and metastatic spread to distant organs as well as treatment responses. In addition to host-derived cfDNA, changes in cancer cells can be detected from cell-free, circulating tumor DNA (ctDNA) by monitoring DNA mutations carried by cancer cells. Here, we will discuss computational approaches to identify and validate robust biomarkers of changed tissue homeostasis using cell-free, methylated DNA in the circulation. We highlight studies performing genome-wide profiling of cfDNA methylation and those that combine genetic and epigenetic markers to further identify cell-type specific signatures. Finally, we discuss opportunities and current limitations of these approaches for implementation in clinical oncology.

## Liquid Biopsies and Cell-Free DNA (cfDNA) in Oncology

**FIGURE 1:**
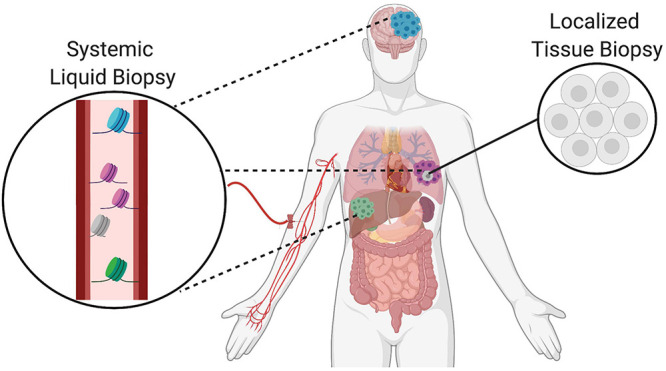
Complementary role of tissue and liquid biopsies in oncology. Localized solid tissue biopsies are invasive and provide a snapshot of limited representational heterogeneity based on the small piece of tissue that is excised. In comparison, liquid biopsies are minimally invasive and allow for serial sampling to provide systemic information about the primary tumor as well as distant metastatic sites indicated in different colors. Thus, liquid biopsies complement tissue biopsies and increase representation of heterogeneity supporting the tracking of clonal evolution over time.

Liquid biopsies are emerging as a minimally invasive approach to complement and potentially advance the traditional standards of care in oncology (Bronkhorst et al., 2019). Tissue biopsies are taken as part of routine clinical care for most solid cancers and used to identify the molecular determinants of disease that can inform both diagnosis and prognosis. However, tissue biopsies are invasive and limited by inaccuracies due to sampling locations, restricted sampling frequency, and poor representation of local tumor heterogeneity as well as dispersed cancerous lesions. To address these limitations, liquid biopsy technologies are rapidly advancing to provide analysis of tumors using circulating biomarkers in fluids such as the blood. One of the main advantages of liquid biopsies is its capacity for serial sampling by simple blood draws. The increased sampling frequency is helpful to monitor clonal evolution of tumor subpopulations as well as to assess evolutionary dynamics influencing treatment response and resistance as well as disease recurrence (Corcoran and Chabner, 2018). Also, liquid biopsies are capable of capturing systemic changes to provide an organism-wide picture of disease progression including the local primary tumor as well as distant metastatic sites and treatment responses across different sites. Finally, liquid biopsies are uniquely able to capture tumor heterogeneity over time, and thus complement traditional tissue biopsies that can only sample locally and at accessible sites (Figure 1).

Similar to tissue biopsies, the major purpose of liquid biopsies in oncology is to identify circulating analytes that provide molecular information about the cancer. In this context, there are a multitude of molecules that may be isolated from biological fluids and targeted for analysis. Until recently, the main focus has been on circulating molecules that can be directly tied back to the primary tumor, including circulating tumor cells (CTCs), cell-free tumor DNA (ctDNA), tumor-educated platelets (TEPs), and tumor secreted vesicles (exosomes, oncosomes, apoptotic bodies) (Best et al., 2015; Rapisuwon et al., 2016). However, as comprehensive approaches gain traction, there has been an expansion to include molecules reflective of dynamic changes to the host, tumor microenvironment and distant metastatic sites as well. Both tumor cells and normal host-derived cells release cell-free DNA (cfDNA) into the circulation as a result of physiological processes. cfDNA is thought to originate from the genomes of dying cells, including cells within tumors, and is reflective of cell turnover rates at steady state as well as altered homeostasis throughout the body with disease (Kustanovich et al., 2019; Heitzer et al., 2020; Rostami et al., 2020). Thus, circulating tumor DNA (ctDNA) is a subset of cfDNA that has different biological characteristics (Table 1). There is still much to be learned about the biology of cfDNA release, distribution, and elimination mechanisms leading to differential stability and circulation half-life in healthy compared to diseased states (Jiang and Lo, 2016; Heitzer and Speicher, 2018; Sanchez et al., 2018; Serpas et al., 2018; Han et al., 2020; Barefoot et al., 2021). The focus of this review will be on methylated cell-free DNA and its utility and applications in cancer diagnosis and management.

**TABLE 1:** Analytes in solid vs. liquid biopsies.

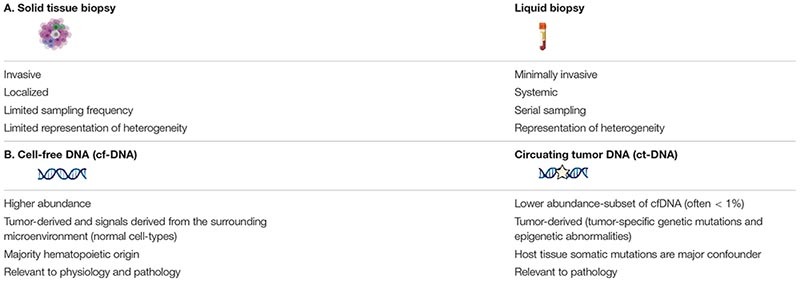

**(A) Comparison of solid and liquid biopsy samples. (B) Comparison of cell-free DNA (cfDNA) and circulating tumor DNA (ctDNA), two circulating analytes found in liquid biopsies.**

## Increased Signal Abundance From Leveraging Epigenetic Changes in Both Tumor and Non-Tumor Cells

**FIGURE 2:**
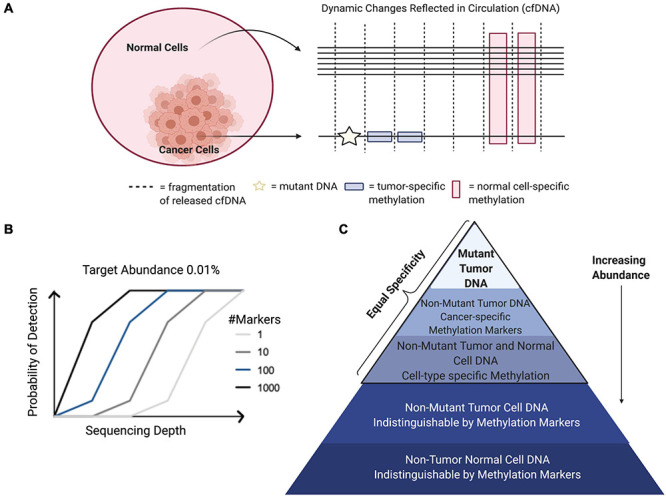
Factors contributing to probability of signal detection. **(A)** Tumors release mutant genomic and epigenomic cfDNA into the circulation. Normal cell types from the surrounding microenvironment and other somatic cells also release cfDNA into the circulation that can be identified through cell-type specific epigenetic markers. Combining tumor cell- and normal host cell-derived signals can increase target abundance in the circulation to increase sensitivity, while maintaining specificity. **(B)** Target abundance, sequencing depth, and breadth of genomic regions assayed are factors that determine signal detection probability in the circulation of cancer patients. **(C)** Relative abundance of cfDNA populations and associated specificity.

There are still many challenges to overcome before liquid biopsies may be routinely implemented in the clinic. Signal abundance (fraction of target cfDNA relative to total cfDNA), sequencing depth, and breadth of genomic regions assayed by sequencing are factors that must be considered to detect signals in the circulation of cancer patients relevant to inform care (Figure 2B; Im et al., 2020). Strategies aimed at increasing any of these factors will improve the odds that informative signals can be detected. Signal abundance is largely a byproduct of the biology of the disease in question and therefore little can be done to modify this variable (Heitzer et al., 2019). For instance, ctDNA is highly correlated with tumor burden, with larger amounts of ctDNA found in the circulation of individuals at advanced stages of tumor progression. For this reason, mutation analysis of ctDNA is limited in its capacity to detect cancer-related signals, especially with low-volume tumors at early stage and relapse (Im et al., 2020). However, signal abundance can be increased by leveraging signals from all cfDNA molecules rather than the smaller subset of fragments containing specific tumor-related mutations (Figures 2A,C). This can be accomplished by targeting tumor-specific epigenetic changes that occur early on during carcinogenesis and thus are found at higher abundance in early stage cancers than tumor-related mutations (Snyder et al., 2016; Ulz et al., 2016; Wong et al., 2016; Leygo et al., 2017; Jiang et al., 2018, 2019; Cristiano et al., 2019; Gai and Sun, 2019; Ivanov et al., 2019; Panagopoulou et al., 2019; Sun et al., 2019; Van der pol and Mouliere, 2019; Sadeh et al., 2021). Further, combining tumor-cell derived signals with those from the surrounding host microenvironment can increase signal abundance (Hoadley et al., 2018; Liu et al., 2018; Haigis et al., 2019; Lam et al., 2019). Tumor DNA identified by genetic or epigenetic markers circulates admixed with non-tumor DNA. The same DNA sequence is found in all non-tumor cells and simple sequence analysis cannot be used to distinguish its cell-type origin. However, covalent and non-covalent epigenetic marks are pivotal to cell-type identity and can be used to distinguish tumor as well as non-tumor DNA, expanding the reach of molecules targeted to reflect disease-pertinent changes (Barefoot et al., 2021).

## Toward “Third-Generation” Liquid Biopsies: From Targeted to Comprehensive Approaches

Despite its highly fragmented nature, advances in sequencing technologies have made comprehensive profiling of low integrity cfDNA possible. At a fixed target abundance and coverage, detection probabilities can be increased by broader sequencing, increasing the number of potential markers assayed (Im et al., 2020). Genomic analysis of ctDNA has decreased sensitivity relative to epigenetic approaches because of lower abundance at any one given marker (Leygo et al., 2017). Increasing the number of potential mutations assayed with whole-genome Next-Generation-Sequencing (NGS) applications has been shown to increase sensitivity, but there can still be a lack of sufficient markers when limited to tumor-specific mutations alone (Im et al., 2020). Comprehensive epigenetic profiling of tumor and non-tumor cfDNA has led to advances in detection of brain cancers, including gliomas, which “hide” behind the blood-brain barrier and have restricted access to release ctDNA into the circulation (Li et al., 2020; Nassiri et al., 2020). In these cases, integration across multiple markers allows for unparalleled sensitivity that cannot be achieved from low numbers of select targeted loci, despite high specificity and deep sequencing. Detection methods trending toward broader sequencing have been termed “third-generation” liquid biopsies and are emerging to allow for more comprehensive assessment of a multitude of signals.

Comprehensive sequencing approaches have unleashed the potential of liquid biopsies to achieve optimal sensitivity; however, there is still a need to improve the specificity and biological relevance of these assays. With the transition from targeted to comprehensive approaches come decreasing signal-to-noise ratios and new challenges to separate true biological signals from background sources of error (Ko et al., 2018). Physiological flux due to clonal hematopoiesis, inflammation, exercise, and other biological factors may dilute out relevant signals and calls for an increased understanding of the mechanisms of cell-free DNA release into the circulation and the distinct processing (Kustanovich et al., 2019; Barefoot et al., 2021). The predominating hematopoietic origins of cfDNA in healthy individuals makes it essential to identify markers separating cell-types of interest from peripheral immune cells (Barefoot et al., 2021). Machine-learning algorithms and data-science-driven approaches are being developed in tandem to reduce dimensionality and make sense of the data available to identify applicable information that may better inform clinical courses of action (Ko et al., 2018). As these approaches become increasingly complex, prior knowledge about the relevance of the features selected will be imperative to maintain biological interpretability.

## Biological Relevance of Cell-Free DNA Methylation Patterns

**FIGURE 3:**
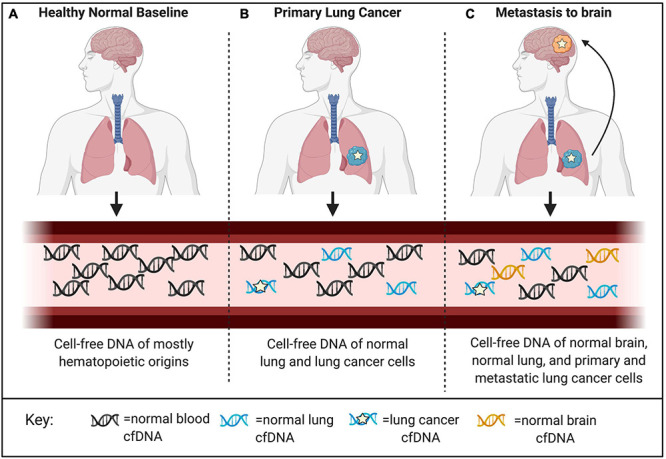
Applications for detection and localization of metastasis and Cancer of Unknown Primary (CUP). **(A)** CfDNA in healthy individuals is mostly of hematopoietic origin. **(B)** The composition of cell-free DNA changes with disease. In this example, primary lung cancer results in increased levels of ctDNA identified by tumor-specific genomic and epigenomic markers, as well as increased levels of cfDNA from the surrounding lung microenvironment identified by normal cell-specific epigenetic markers. **(C)** Genomic mutations occur independently in primary and metastatic tumor sites. Liquid biopsies are capable of capturing this heterogeneity; however, mutations alone cannot localize these clonal populations to their tissue origins at the primary tumor site and distant metastatic site. As a complementary approach, normal tissue- and cell-type epigenetic markers can be used for detection and localization of metastasis and Cancers of Unknown Primary (CUP).

DNA methylation functions as an epigenetic regulatory mechanism and involves covalent addition of a methyl-group to the 5-carbon of cytosine (5mc). DNA methylation occurs most commonly in the context of CpG dinucleotides (Greenberg and Bourc’his, 2019). One of the main benefits to harnessing cell-free methylated DNA for liquid biopsy applications in cancer is the potential to exploit prior knowledge about the biological relevance of these marks. DNA methylation is an intrinsic mark of cell identity and pathologic alterations of DNA methylation are hallmarks of cancer (Greenberg and Bourc’his, 2019). The DNA methylation landscape changes in a highly regulated manner throughout development. Before embryo implantation, there is a global erasure of DNA methylation that is reset in multiple stages leading to the creation of cell-type specific methylation patterns, paralleling ongoing cell differentiation and organogenesis (Dor and Cedar, 2018). Once established, this pattern of DNA methylation is highly stable and conserved across DNA replication, making DNA methylation the predominant mechanism for inherited cellular memory during cell growth (Daniūnaitė et al., 2019). DNA methylation patterns may be selected as features that are relatively hyper- or hypo-methylated in specific cell types or in the context of specific cancers. Therefore, while there has been extensive characterization of DNA methylation changes that occur with disease and physiological aging, these changes occur only at specific locations throughout the epigenome allowing methylation states at regions critical to cell-type identity to remain constant over time (Michalak et al., 2019). This stability allows methylated cfDNA to serve as a robust biomarker in the face of patient heterogeneity, capable of being generalized across diverse patient populations (Dor and Cedar, 2018). There are many areas where liquid biopsies can be applied in clinical oncology. These include, but are not limited to, efforts aimed at early detection, assessment of prognosis, detection of minimal residual disease, metastasis, targeted-therapy selection, and treatment response monitoring (Sina et al., 2019; Luo et al., 2021). Both cancer and cell-type specific cell-free DNA methylation markers have been employed in each of these applications; however, there are important distinctions based on using disease-specific or normal cell-type specific markers that are worth noting. Specifically, cell-type specific DNA methylation markers have unique applications to localize cancers of unknown primary (CUP) as well as to detect metastases (Figure 3; Gai et al., 2018; Moss et al., 2018). In addition, systemic therapy-related adverse event monitoring remains one of the most promising applications.

## Cell-Free DNA Methylation Technologies

**FIGURE 4:**
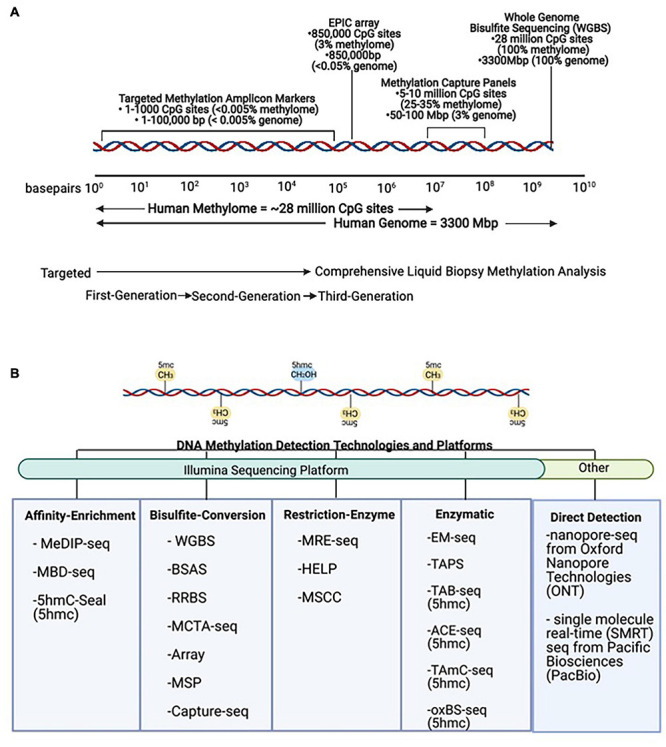
DNA methylation technologies and platforms for signal detection. **(A)** Scale representation of DNA methylation technologies from targeted First- and Second-Generation toward comprehensive Third-Generation applications in liquid biopsies. **(B)** Methods for detection of DNA methylation. The same DNA sequence is found in all non-tumor cells, and simple sequence analysis cannot be used to distinguish its cell-type identity. However, these methods can be used to detect DNA methylation (5mc) and DNA hydroxymethylation (5hmc) levels in tumor and non-tumor cells. MeDIP-seq, Methylated DNA immunoprecipitation sequencing; MBD, methyl-CpG-binding domain sequencing; WGBS, Whole Genome Bisulfite Sequencing; BSAS, Bisulfite Amplicon Sequencing; RRBS, Reduced Representation Bisulfite Sequencing; MCTA-seq, methylated CpG tandem amplification and sequencing; MSP, Methylation Specific PCR; MRE-seq, methylation-sensitive restriction enzyme sequencing; HELP, Hpall-tiny fragment enrichment by ligation-mediated PCR; MSCC, Methyl-sensitive Cut Counting; EM-seq, Enzymatic Methyl-Sequencing; TAPS, TET-assisted pyridine borane sequencing; TAB-seq, TET-assisted bisulfite sequencing; ACE-seq, APOBEC-coupled epigenetic sequencing; hmc-CATCH, chemical-assistant C-to-T conversion of 5hmC sequencing; oxBS-seq, oxidative bisulfite sequencing.

There are many techniques that can be used to study DNA methylation as well as different strategies that can be applied to classify and quantify methylation status (Olkhov-Mitsel and Bapat, 2012; Kurdyukov and Bullock, 2016; Galardi et al., 2020; Zhao et al., 2020). These methodologies must be able to distinguish between methylated and unmethylated cytosines. This review mainly focuses on 5mc as it is the most commonly characterized epigenetic mark in cancer. However, other DNA modifications, including 5-hydroxymethylcytosine (5hmc), are thought to be more dynamic, reflecting active demethylation events, and may be complementary to characterize as well (Song et al., 2017). The different DNA methylation detection technologies and platforms are categorized in Figure 4. The main methods are restriction enzyme digestion, affinity enrichment, bisulfite-conversion, and enzymatic modification approaches. To date, several of these approaches have been successfully implemented to study genome-wide cfDNA methylation, highlighted in Table 2. Restriction enzyme-based methods cleave DNA at enzyme specific CpG sites. However, the highly fragmented nature of cfDNA and limited frequency of CpG-containing recognition sites make this approach challenging for comprehensive profiling of cfDNA (Huang and Wang, 2019). cfMeDIP-seq is an affinity-based approach that enriches for methylated DNA using 5mc-specific antibodies (Shen et al., 2018). As such, it is capable of characterizing overall methylation levels across a region, but not at single CpG sites. In addition, the majority of cell-type specific methylation markers in the human body are hypomethylated as a result of methylation resetting that takes place throughout tissue differentiation and development. These methods that specifically enrich for hypermethylated DNA may have limited detection potential at these regions of interest.

**TABLE 2:** Feasibility of tissue-of-origin analysis in oncology using cell-free DNA methylation markers.

**Disease**	**Methylation data type**	**Marker type**	**Deconvolution method**	**Publication**
HCC, NIPT, Transplant	WGBS	Tissue-specific	QP	Sun et al., 2015
PDAC, CRC, Diabetes, Transplant, MS, TBI, IBD	BSAS	Tissue-specific	Read-specific binary classification	Lehmann-Werman et al., 2016, 2018
Transplant	WGBS	Tissue-specific	QP	Cheng et al., 2017
CRC, LCP	RRBS, WGBS	Both	Multi-class prediction, RF, feature extraction “haplotype blocks”	Guo et al., 2017
MI, sepsis	BSAS	Tissue-specific	Read-specific binary classification	Zemmour et al., 2018
CRC, BRCA, PDAC, CUP, Transplant, Sepsis	450K array	Tissue-specific	NNLS regression	Moss et al., 2018
Transplant, infection	WGBS	Tissue-specific	QP	Cheng A. P. et al., 2019
Neurotrauma + neurodegenerative disease	tNGBS (multiplex 35 amplicons)	Tissue-specific	Read-specific binary classification (k-mer analysis)	Chatterton et al., 2019
HCT, GVHD, transplant	WGBS	Tissue-specific	QP	Cheng et al., 2020
HCC, cirrhosis, cholelithiasis, acute pancreatitis	MCTA-seq	Tissue-specific	PSO	Liu Y. et al., 2019
BRCA	BSAS	Tissue-specific	Read-specific binary classification	Moss et al., 2020
mCRPC	Cpature-seq/WGBS	Both	PCA	Wu et al., 2020
12 cancer types	Cpature-seq/WGBS	Both	Ensemble logistic regression	Liu et al., 2020
ALS, pregnancy	WGBS	Tissue-specific	Bayesian EM algorithm (CelFiE) likelihood-based	Caggiano et al., 2020
Transplant, AKI	cfNOME-seq	Tissue-specific	LSM (QP)	Erger et al., 2020
COVID-19	WGBS	Tissue-specific	NNLS regression	Cheng et al., 2021
HCC, CRC, LCP	WGBS	Cancer-specific	Read-specific, likelihood-based	Kang et al., 2017; Li et al., 2018
LCP, HCC. PDAC, GBM, CRC, BRCA	hMe-Seal (5hmc)	Cancer-specific	RF, Mclust	Song et al., 2017
PDAC, AML, BRCA, CRC, RCC, PLC	MeDIP-seq	Cancer-specific	Limma, binomial GLM	Shen et al., 2018
Pediatric MB	WGBS/CMS-IP-seq	Cancer-specific	Multivariate Cox regression linear model	Li et al., 2020
Glioma, intracranial tumors	MeDIP-seq	Cancer-specific	Binomial RF	Nassiri et al., 2020

**HCC, Hepatocellular Cancer; NIPT, Non-Invasive Prenatal Testing; PDAC, Pancreatic Cancer; CRC, Colorectal Cancer; MS, Multiple Sclerosis; TBI, Traumatic Brain Injury; IBD, Inflammatory Bowel Disease; LCP, Lung Cancer Primary; MI, Myocardial Infarction; BRCA, Breast Cancer; CUP, Cancer Unknown Primary; GBM, Glioblastoma Multiforme; AML, Acute Myeloid Leukemia; RCC, Renal Cell Carcinoma; HBC, Hepatobiliary Cancer; NSCLC, Non-Small Cell Lung Cancer; HCT, Hematopoietic Cell Transplant; GVHD, Graft-vs.-Host Disease; AKI, Acute Kidney Injury; ALS, Amyotrophic Lateral Sclerosis; MB, Medulloblastoma; WGBS, Whole Genome Bisulfite Sequencing; BSAS, Bisulfite Amplicon Sequencing; RRBS, Reduced Representation Bisulfite Sequencing; ddPCR, Droplet Digital PCR; tNGBS, targeted Next Generation Bisulfite Sequencing; MeDIP-seq, Methylated DNA immunoprecipitation Sequencing; CMS-IP-seq, Cytosine 5-methyenesulphonate-immunoprecipitation sequencing; MCTA-seq, Methylated CpG Tandems Amplification Sequencing; cfNOME-seq, cell-free Nucleosome Occupancy and Methylation Sequencing; RF, random forest; GLM, generalized linear model; NNLS, Non-Negative Least Squares; LSM, Linear Least Squares Minimization; QP, Quadratic Programming; PSO, Particle Swarm Optimization; EM, Expectation-Maximization. Some of the materials are based on Barefoot et al. (2021).**

Bisulfite conversion chemically modifies DNA so that unmethylated cytosines (C) are deaminated to uracil (U) to be later replaced by thymine (T) via PCR, while unmethylated cytosines are protected and remain cytosine (C) (Olova et al., 2018). The majority of comprehensive cfDNA methylation profiling has been done using bisulfite conversion methods, including Whole Genome Bisulfite Sequencing (WGBS), Reduced Representation Bisulfite Sequencing (RRBS), Methylated CpG Tandem Amplification and Sequencing (MCTA-seq), and Methylation Arrays. WGBS and RRBS are capable of detecting DNA methylation at single-base resolution. More importantly, these methods are capable of detecting read-specific DNA methylation patterns (Scott et al., 2020). WGBS is the most comprehensive approach, but it can be costly to sequence the whole genome to an informative depth. However, sequencing costs are decreasing, making this approach more attractive. RRBS has been optimized in a few instances for accommodating highly fragmented cfDNA molecules (Guo et al., 2017; De Koker et al., 2019). Despite these modifications, the use of restriction enzymes in this sequencing approach give rise to the same limitations as restriction-enzyme based methods. MCTA-seq uses primers to preferentially amplify methylated CpG islands (CpG tandem regions) and, while being more targeted, this approach is also biased toward hypermethylated regions (Liu X. et al., 2019). Methylation hybridization arrays allow for single-base resolution but do not allow for pattern analysis of multiple CpG sites from the same molecule and have reduced genome-wide coverage of CpG sites compared to NGS approaches (Moss et al., 2018).

While bisulfite conversion has long been considered the gold standard of methylation detection, there are major limitations that recent advances in enzymatic approaches show promise in overcoming (Schutsky et al., 2018; Liu Y. et al., 2019). For instance, sodium bisulfite is a harsh chemical treatment that causes unwanted DNA degradation and fragmentation, resulting in uneven genome coverage. Enzymatic Methyl-seq (EM-seq) uses the enzyme APOBEC to deaminate unmethylated cytosines and protects methylated cytosines from conversion by utilizing TET2 as an oxidative enhancer (Vaisvila et al., 2019). This results in the same base conversions as bisulfite sequencing, but this method has been shown to cause less DNA damage and as a result is more sensitive, requiring smaller amounts of input DNA. This method is used in a recent publication to profile cytosine methylation and nucleosome occupancy at the same time, a feat made possible from retention of the original cfDNA structure without fragmentation or degradation (Erger et al., 2020).

The nuances of these different methodologies to detect DNA methylation make choosing the right method and accounting for its limitations essential toward accurate interpretation of results. Methylation detection technologies are rapidly evolving, leading to expanded potential applications. For instance, one such advancement involves the direct detection of methylation without treatment of DNA, possible with nanopore-sequencing from Oxford Nanopore Technologies (ONT) and single molecule real-time (SMRT) sequencing from Pacific Biosciences (PacBio) (Flusberg et al., 2010; Liu Q. et al., 2019; Ewing et al., 2020; Yuen et al., 2020; Tse et al., 2021). Although direct detection of methylation is not currently possible with cfDNA inputs, these advances point toward new possibilities in the future.

## Tissue-Of-Origin (TOO) Deconvolution Analysis: Using Healthy Cell-Type Signals to Inform About Disease

**FIGURE 5:**
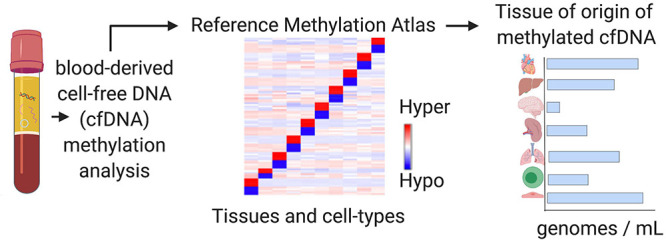
Tissue-of-origin deconvolution analysis. CfDNA is a mixture of fragments released from healthy and diseased cells in different tissue types throughout the human body into the circulation. DNA methylation is highly cell-type specific and can be used to identify the cellular origins of cfDNA at specific markers. Tissue-of-origin (TOO) analysis traces cfDNA molecules back to the tissues and cell types they originated from and use changing tissue proportions to reveal altered tissue homeostasis in diseased states or during therapy.

Tissue-of-origin (TOO) analysis takes each individual cell-free DNA molecule in the circulation and routes it back to its tissue and cellular origins as a non-invasive monitoring tool for tissue damages (Figure 5). At steady state, cfDNA is released into the circulation reflective of cellular turnover happening throughout the human body, resulting in a complex mixture of fragments (Barefoot et al., 2021). On average, the plasma from healthy individuals has 1,500 genome equivalents or roughly 10 ng/mL cfDNA concentration (Moss et al., 2018). With cancer, cfDNA levels are thought to increase in parallel with disease progression as a result of increased proliferation and death rates of tumor cells (Kustanovich et al., 2019). However, relying on concentration of cfDNA alone to diagnose disease is too simplistic of an approach, as concentration is not an absolute indicator of disease and changes can result from a plethora of factors, including exercise, inflammation, and induction of cellular senescence. Detection of changing cell-type proportions from alterations in cfDNA composition is a more reliable approach. Shifting cfDNA makeup has been used for monitoring altered death rates of cells in different tissues, applicable to a broad spectrum of physiological and pathological conditions as well as therapeutic interventions. These include non-invasive prenatal testing, solid organ transplant, cancer, neurodegenerative and autoimmune pathologies, among many others (Sun et al., 2015; Zemmour et al., 2018; Chatterton et al., 2019; Cheng A. P. et al., 2019). To demonstrate feasibility, DNA methylation patterns specific to a variety of epithelial, endothelial, nervous, stromal, muscle, fat, and immune cell-types have been discovered and successfully applied using TOO analysis of cfDNA (Lehmann-Werman et al., 2016, 2018; Moss et al., 2018). In addition to tumor-derived DNA, changes to the host microenvironment can contribute to altered cell-type proportions of cfDNA in the circulation through cancer-related changes to normal tissue architecture. Although normal cell-specific DNA methylation markers are used, relevance to disease is inferred through abnormal detection in the circulation as a result of aberrant cell death and tissue damages (Heitzer et al., 2020). Thus, the changing proportion of normal cell types found in the circulation can be used to inform about disease states (Houseman et al., 2012; Teschendorff et al., 2017; Zheng et al., 2018; Huang et al., 2019; Barefoot et al., 2021). Recent studies demonstrating the feasibility of using cell-type specific cfDNA methylation marks for TOO analysis in cancer are described below (Table 2). This methodology is useful to detect damage to specific cell types in tissues and has many applications to inform diagnostics in the clinic as well as to reveal complexities of cancer pathophysiology at the cellular level.

## Computational Methods for Cell-Mixture Deconvolution in Liquid Biopsy

Advances in liquid biopsy technology have led to the generation of massive amounts of methylation sequencing data that can be difficult to analyze due to the extensive number of possible features in the human methylome. Computational methods, including many machine learning techniques, have been developed to better handle such data by isolating specific signals and discriminative features, thereby reducing the dimensions of the data so that it is easier to interpret (Ko et al., 2018). In this dimension reduction approach, the data is projected into lower-dimensional spaces, ultimately with the aim to improve prediction accuracy through increasing the signal-to-noise ratio. As previously described, the total makeup of cfDNA can be modeled as a complex mixture with TOO deconvolution analysis aiming to trace each individual cfDNA molecule back to its cellular origins as a non-invasive measure of tissue damage.

There are many computational methods that have been successfully applied to facilitate TOO deconvolution of cfDNA (Table 2). These include reference-based supervised learning models, which utilize labeled training and test datasets for classification tasks (Teschendorff et al., 2017). Commonly used methods include linear or logistic regression and random forests. In addition, one study uses Particle Swarm Optimization as a supervised global optimization method. While global optimization methods may be supervised, semi-supervised, or unsupervised, in this case the method is applied as a supervised learning model (Liu Y. et al., 2019). There are also several unsupervised learning models, including clustering and density estimation methods, in which the goal is to learn the inherent structure and relations of unlabeled data (Houseman et al., 2016). One advantage of unsupervised, reference-free algorithms is the ability to estimate contributions from unknown cell types, or cell types for which reference methylation data is not available. However, the biological meaning of the features selected in these models is often lost or difficult to interpret, making it more challenging to explain the relevance of results. Recently, deep learning has also been applied as a powerful modeling technique for deconvolution of DNA methylation data as these methods perform simultaneous feature extraction and classification (Levy et al., 2020; Menden et al., 2020). As a high-level overview, the computational methods for cell-mixture deconvolution can be generalized as adhering to the following format that is consistent across liquid biopsy applications. Initially, features are selected or extracted that can characterize variation among cell-type contributors in the circulation. Then, statistical models are built to estimate the mixing proportions of each cell type based on the reduced number of discriminative DNA methylation features selected. Typically, these models are trained using reference data where the mixing proportions are already known and then tested on datasets where the mixing proportions are unknown for evaluation (Feng et al., 2019). As a final step, predictive models can be developed after deconvolution, using the inferred cell-mixture proportions as predictors to estimate disease phenotypes.

Despite demonstrated success applying these computational models to cfDNA methylation deconvolution, these algorithms were originally designed to be learned from very large training datasets (Ko et al., 2018). In order to maintain predictive capabilities, modifications are necessary to optimize these models for working with smaller and often more diverse datasets, typical to the field of liquid biopsy. With this in mind, there are important biological properties of cell-free DNA methylation that can be leveraged toward this goal. First, in comparison to DNA in tissues that is artificially sheared for introduction to standard library preparation methods, the fragmentation patterns of cfDNA are biologically derived (Lo et al., 2021). The majority of cfDNA fragments are ∼167 bp, representing the length of DNA wrapped around a nucleosome and reflective of degradation by nucleases as a by-product of cell death. This fragmented nature of cfDNA lends itself to methods developed for characterizing cfDNA at the level of single molecules as opposed to population-level averages at single CpG sites (Li et al., 2018). Read-specific analysis allows for each read-pair to be modeled as an independent sample reflective of each individual cfDNA molecule. This allows the depth of sequencing to be utilized toward increasing sample size (Scott et al., 2020). In addition, the density of neighboring CpG sites varies across the human genome with highly dense organization defined as CpG islands. Methylation status at adjacent CpG sites is co-regulated in CpG islands due to the expanse of methylating and demethylating enzymes acting in the area (Marzese and Hoon, 2015). This co-dependency can be leveraged to increase specificity through modeling the methylation features selected with pattern analysis. DNA methylation detection technologies and computational approaches that take advantage of pattern analysis of individual cfDNA molecules have demonstrated to be more robust and to increase the sensitivity and specificity of cell-type proportion estimations (Lehmann-Werman et al., 2016; Guo et al., 2017; Li et al., 2018; Zemmour et al., 2018; Chatterton et al., 2019). Overall, the biological relevance of selected DNA methylation markers and derived tissue proportions to disease can be utilized to inform analysis and model optimization.

## Combining Genetic and Epigenetic Markers to Non-Invasively Monitor Treatment Response and Therapy-Related Adverse Events

**FIGURE 6:**
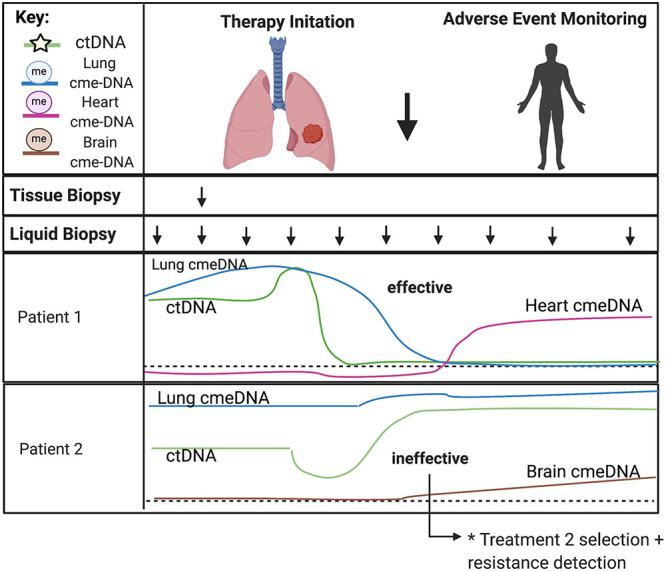
Predicting treatment response and therapy-related toxicities from combined genetic and epigenetic analyses of cfDNA. The minimally invasive nature of liquid biopsies allows for serial sampling to monitor changes over time, especially under selective pressures from ongoing therapy. CtDNA can be used to track clonal heterogeneity over time to assess treatment response and detect treatment-resistant clones. Normal cell-specific cfDNA methylation patterns can be used in combination with ctDNA to assess the impact of treatment to the surrounding tumor microenvironment and to monitor for therapy-related toxicities in somatic cell-types. Acronyms: ctDNA, (circulating tumor DNA); cme-DNA, (circulating methylated cell-free DNA).

There is a need to identify predictive biomarkers for real-time monitoring of therapy-related adverse events relative to therapeutic efficacy. Combining changes to mutant ctDNA with altered proportions of cell-type specific cfDNA can reflect intervention-based changes (Figure 6; Erger et al., 2020; Guo et al., 2020; Wu et al., 2020). Therapy regimens for many cancers involve surgery, chemotherapy, radiotherapy, targeted therapy, and immunotherapy (Hofman et al., 2019). Each of these interventions can have a different systemic effect, and the ability to distinguish different cell types participating and potentially contributing to toxicities with cfDNA in serially drawn blood samples could significantly impact therapeutic decision making. Although imaging modalities can be used as an indirect way to gauge therapeutic efficacy, these results are often unreliable and difficult to interpret. Imaging results can be clouded by depictions of pseudoprogression, making them ineffective or crude instruments to monitor for concurrent changes necessary to guide therapy decisions (Maia et al., 2020). In contrast, the half-life of cfDNA is between 15 min and 2 h (Khier and Lohan, 2018). The rapid clearance allows for serial analysis of disease evolution over time, especially under selective pressures from ongoing therapy (O’Leary et al., 2018; Oellerich et al., 2019; Nabet et al., 2020; Peter et al., 2020). This technology allows for serial sampling to include a baseline comparison from which therapy-related relative changes may be assessed, taking into account patient specific co-morbidities at an individualized level.

Combining genetic and epigenetic analyses of cell-free DNA has many unique advantages when applied to precision therapeutics in cancer (Cheng T. H. et al., 2019; Zhang et al., 2019). Liquid biopsies have been shown to accurately characterize tumor genotypes and allow for molecular subtype classification to provide a comprehensive view of intratumor heterogeneity (Cohen et al., 2018; Christensen et al., 2019; Heitzer et al., 2019). High sampling frequency allows for modeling of evolutionary dynamics of tumor progression. Also, molecular changes identified after initiation of therapy can provide insight into therapy response as well as track tumor subclones that may lead to emergence of therapy resistance (Ahronian and Corcoran, 2017; Zhou et al., 2020). The systemic view provided by serial liquid biopsies is ideal to monitor widespread changes that may better inform clinical decision making in the face of uncertainty. For example, in the case of surgical removal of the tumor or therapeutic success, liquid biopsies can be used to monitor for minimal residual disease and recurrence. While ctDNA can be used to track molecular changes in the circulation, there is a benefit to monitoring the cancer-related changes to the host microenvironment in tandem requiring a combined genetic and epigenetic analysis. Cell-specific cfDNA methylation patterns of normal cells can be used in combination with ctDNA to assess the impact of treatment also on the surrounding tumor microenvironment. This is particularly useful to surveil for metastatic disease in distant tissue types from the primary tumor as well as to monitor for therapy-related toxicities in somatic cell types (Zhang et al., 2019). Further, liquid biopsies can help delineate factors that underlie clinical outcomes, providing a basis for recommending different treatments based on anticipated benefit to the patient. Liquid biopsies can identify predictive biomarkers to guide selection of treatment, recognize off-target effects, and develop individualized treatment plans for patients (Hofman et al., 2019). These applications provide a more complete picture of therapeutic response as well as tissue-specific cellular toxicity to better inform clinical care and management throughout the treatment process.

## Future Directions and Conclusion

Liquid biopsies are rapidly emerging as an alternative and complementary approach to traditional solid tissue biopsies and have high utility for many applications in clinical oncology. Technology advances have made genome-wide profiling of circulating analytes possible and allow for transition from targeted to comprehensive approaches. With transition to “third-generation” liquid biopsies, DNA methylation patterns can be used to leverage signals from both tumor and non-tumor cells to increase signal abundance and discern biological relevance (Im et al., 2020). Despite great potential, comprehensive applications of liquid biopsy in oncology are still in their infancy. Additional large-scale, stratified, and randomized longitudinal studies are needed to begin to understand the complex interactions and biological significance of the comprehensive data identified from NGS technologies. Future work aimed at elucidating the biology of cell-free DNA release is needed to begin to control for co-morbidities and other confounding variables. Efforts aimed at assessing the effect of therapy regimens (chemo, radiation, immunotherapy, etc.) on tumor and non-tumor signals will become essential to determine what signals can be derived from the circulation. Tissue-of-origin analysis can be used to localize signals and generation of cell-type specific reference methylomes can improve specificity of features selected for application of TOO analysis in cancer (Moss et al., 2018; Barefoot et al., 2021). In addition, combining genetic and epigenetic markers may improve targeted-therapy selection and treatment response monitoring. These approaches are potentially synergistic, and future integration of signals across multiple genetic and epigenetic omics levels could fine-tune these applications for optimal use in precision oncology.

## Author Contributions

MB and AW wrote the first draft of the manuscript. All authors contributed to manuscript revision, read, and approved the submitted version.

## Conflict of Interest

Georgetown University filed a patent related to some of the approaches described in this manuscript. MB and AW are named as inventors on this application and declare that as a potential conflict of interest. The remaining authors declare that the research was conducted in the absence of any commercial or financial relationships that could be construed as a potential conflict of interest.

## Publisher’s Note

All claims expressed in this article are solely those of the authors and do not necessarily represent those of their affiliated organizations, or those of the publisher, the editors and the reviewers. Any product that may be evaluated in this article, or claim that may be made by its manufacturer, is not guaranteed or endorsed by the publisher.
